# Ethnic group differences in obesity in Asian Americans in California, 2013–2014

**DOI:** 10.1186/s12889-021-11612-z

**Published:** 2021-08-25

**Authors:** Shaoqing Gong, Kesheng Wang, Ying Li, Zhongliang Zhou, Arsham Alamian

**Affiliations:** 1 School of Public Policy and Administration, Xi’an Jiaotong University, Xi’an, Shaanxi Province China; 2 Department of Family and Community Health, School of Nursing, West Virginia University, Morgantown, West Virginia USA; 3 Department of Environment Health, College of Public Health, East Tennessee State University, Johnson City, TN USA; 4 School of Nursing and Health Studies, University of Miami, Coral Gables, Florida USA

**Keywords:** Obesity, Asian Americans, Ethnicity, Health disparity

## Abstract

**Background:**

Obesity has been generally understudied in Asian Americans. It is important to identify subgroups of Asian Americans at high risk of obesity to help develop targeted interventions for those subgroups. This study aimed to examine the disparities in obesity among Asians (i.e., Chinese, Filipino, Japanese, Korean, and Vietnamese) living in California.

**Methods:**

A sample of Adult Americans in California (*n* = 47,970) including Asian American adults (*n* = 3810) aged 18 years or older were obtained from the 2013–2014 California Health Interview Survey (the U.S. nation’s largest state cross-sectional health survey). Body mass index was calculated using self-reported height and weight. Weight status was determined using the WHO Asian BMI cut points in 4 categories: < 18.5 kg/m^2^ (underweight), 18.5–22.9 kg/m^2^ (normal weight), 23–27.5 kg/m^2^ (overweight), and ≥ 27.5 kg/m^2^ (obese). Multiple logistic regression analyses were used to estimate odds ratio (OR) and 95% confidence interval (CI) after adjustment for covariates.

**Results:**

Overall, the prevalence of Asians was 23.3% for obesity and 40.0% for overweight. The obesity prevalence was higher in Asians who were males, aged 45–64 years old, had higher family income, were current smokers, never got married, had lower education level, had an insufficient level of physical activity, and had more frequent consumption of fast foods. After adjusting for other factors, compared to Whites, being Hispanics and Blacks were associated with higher odds of obesity (OR = 1.47, 95%CI = 1.31–1.65; OR = 2.04, 95%CI = 1.65–2.53, respectively); being Chinese, Korean, and Vietnamese were associated with lower odds of obesity (OR = 0.28, 95%CI = 0.18–0.45; OR = 0.14, 95%CI = 0.04–0.46; OR = 0.28, 95%CI = 0.14–0.58, respectively). Compared to Chinese, being Japanese and Filipino were associated with higher odds of obesity (OR = 2.75, 95%CI = 1.52–4.95; OR = 2.90, 95%CI = 1.87–4.49, respectively).

**Conclusions:**

The prevalence of adult obesity was high among Asian Americans in California. Ethnic/racial disparities in obesity among Asian Americans in California were observed in 2013–2014. Compared to Whites, being Chinese, Korean, Vietnamese were associated with lower odds of obesity. Among Asians, compared to Chinese, being Japanese and being Filipino were associated with higher odds of obesity. These findings can help design better interventions to reduce racial and ethnic disparities in obesity, especially for Asian Americans.

## Background

Research at the national level in the United States (U.S.) has shown a significant increase in obesity prevalence, particularly over the past two decades [1]. In California, there were more than 7 million obese adults and adolescents in 2011–2012. Overall, the prevalence of obesity in adults has increased by approximately 32% from 2001 (19%) to 2011–2012 (25%). The increase in the prevalence of obesity has been observed in Latino, White, African-American, and Asian adults during this period. Moreover, compared to Whites in California, the prevalence of obesity was higher among African-Americans, American Indians, and Latinos [2]. Obesity has been identified as a major risk factor for multiple chronic conditions such as diabetes and cardiovascular disease [3, 4]. However, important variation among racial/ethnic subgroups would be ignored if we only focus on the overall population. It has been suggested that the prevalence of and influence of obesity among subpopulations are very different according to races/ethnicities [5].

Some racial/ethnic groups have been more influenced by obesity than others. In the U.S., from 2011 to 2012 to 2017–2018, the obesity prevalence was highest in Non-Hispanic Blacks (from 48.1 to 49.6%), followed by Hispanics (from 42.5 to 44.8%), non-Hispanic Whites (from 34.5 to 42.2%), and non-Hispanic Asians (from 11.7 to 17.4%) [6, 7]. National Surveys [8] show that Asian subgroups have obvious ethnic differences and dramatic increases in the prevalence of obesity, suggesting that these subgroups warrant increased monitoring of obesity and its related risk factors. In California, racial/ethnic differences in body mass index (BMI) were also observed among adults for both sexes [9]. The reasons of racial/ethnic disparities in obesity have been explored and have focused on the important roles of a variety of socioeconomic, demographic, and behavioral characteristics [10]. For example, compared to Californian Blacks and Hispanics, Californian Whites had lower poverty rates and higher education levels. However, the reason as to why Asians have lower BMI rates than Whites with adjustment for covariates remains unclear [9]. A number of studies have examined the sex and racial/ethnic disparities in obesity [11]. However, evidence is very limited with regard to differences in obesity rates among Asian Americans; further most of the few existing studies used samples that were multiracial, and therefore may not be generalizable to Asian Americans [12]. Due to lack of data or small sample sizes for Asians in most studies of obesity or BMI, Asian Americans were often excluded, or when they were included, they played a small role in study results [9, 13].

However, it is best not to treat Asian Americans as simply one group because they are highly diverse in national origins [14] and socioeconomic status (SES) [15] (e.g., far exceeding national averages for some subgroups). Furthermore, the prevalence of health conditions in Asian adults such as cardiovascular diseases [16, 17] appears different among subgroups. Because of such diversity in multiple dimensions related to obesity, it is important to identify subgroups of Asian Americans at high risk of obesity to help develop targeted interventions for those subgroups.

To address the above-mentioned gaps in the literature, we examined the disparities in the patterning of obesity among Asians (e.g., Chinese, Filipino, Japanese, Korean, and Vietnamese). We also brought insight into the role of a number of characteristics (e.g., age, sex, family income, marital status, education level, physical activity, and fast food consumption) in predicting obesity among Asian Americans. Because there is a large size of Asian population and Asians are highly diverse in California, it is warranted to understand these disparities and the determinants of obesity as well as associated policy challenges. Consequently, public health resources could be aimed at those most at risk.

## Methods

### Participants

A sample of Adult Americans in California (*n* = 47,970) including Asian American adults (*n* = 3810) aged 18 years or older was obtained from the California Health Interview Survey (CHIS) with data pooled from the 2013–2014 survey years. The CHIS is the largest state health survey in the U.S. and provides representative data using a county-based, stratified sampling design to represent California’s non-institutionalized residents in all 58 counties in California. It is a continuous survey since 2001 on a variety of social and environmental factors that may affect health. The CHIS used a dual-frame, multi-stage sample design. The random-digit-dial (RDD) sample included telephone numbers assigned to both landline and cellular service. Details about the sampling design can be found elsewhere [18]. Every sampled adult received a final weight and a set of 80 replicate weights. The final weight accounts for sample selection probabilities and statistical adjustments for potential undercoverage and nonresponse biases. The replicate weights are specially designed for valid variance estimation in the absence of confidential sample design information. When using replicate weights in conjunction with the final weight, the estimates and their variance estimation would be unbiased. The CHIS has conducted interviews in several languages to increase Asian American representation. In addition to English and Spanish, CHIS is administered in four Asian languages: Cantonese, Mandarin, Korean, and Vietnamese.

East Tennessee State University Institutional Review Board approved the secondary analysis of the present study. Participant consent was not necessary as this study involved the use of a previously-published de-identified database.

### Study variables

#### Obesity

BMI was calculated as weight (kg) divided by height squared (m^2^) for self-reported data. Weight status was determined using the World Health Organization (WHO) Asian BMI cut points in Asian groups as 4 categories to account for racial differences in body fat percentage at the same BMI level: < 18.5 kg/m^2^ (underweight), 18.5–22.9 kg/m^2^ (normal weight), 23–27.5 kg/m^2^ (overweight), and ≥ 27.5 kg/m^2^ (obese) [19].

#### Race/ethnicity

We used the self-reported Asian ethnicity variable constructed by CHIS, which includes five categories: Chinese (including Taiwanese), Japanese, Korean, Filipino, and Vietnamese.

#### Covariates

Covariates included age (continuous variable), sex, family income (examined as a percentage of the federal poverty level (FPL), which adjusts for total household income and number of members in the household: below 100, 100 to 299%, or 300% and above of the FPL), smoking status (current smoker, not current smoker), marital status (married, never married, or other), education attainment (high school, college, or graduate), physical activity (walking at least 10 min for leisure past 7 days, walking at least 10 min for transport past 7 days), and fast food consumption (never, 1–2 times, and ≥ 3 times). Fast food consumption was determined by response to the following question: “In the past 7 days, how many times did you eat fast food? Include fast food meals eaten at work, at home, or at fast-food restaurants, carryout or drive through” [20].

### Statistical analysis

The SAS PROC SURVEYFREQ procedure was used to weight and estimate population proportions. The Chi-square test was used to compare the prevalence of the study outcome across age, gender, and races and other factors. Specifically, the prevalence of weight status (underweight, normal weight, overweight, and obesity) was examined according to race/ethnicity (Chinese, Filipino, Japanese, Korean, Vietnamese, Whites, Hispanics, Blacks, and other races) and sex. Demographic and lifestyle characteristics was examined among Californian Asians (i.e., overall, Chinese, Korean, Japanese, Filipino, and Vietnamese). The SAS PROC SURVEYLOGISTIC was used to estimate odds ratios (ORs) and 95% confidence intervals (95% CI) for the relationship between potential risk factors and obesity. First, weighted multiple logistic regressions were used to examine the association between race/ethnicity and obesity among Californian adults. Second, we used weighted multiple logistic regression analyses to examine the association between race/ethnicity and obesity among different subgroups of Asian Americans by sex. Variables with *P* values significant at or below 0.20 in univariate analyses were included in the final multiple logistic regression models. SAS version 9.4 (SAS institute, Cary, NC) was used for analysis and computation of weighted estimates for projection to the California population.

## Results

### Prevalence of obesity in adults of California

Table 1 shows weighted prevalence of weight status in Californian adults by sex and race/ethnicity from 2013 to 2014. Overall, the prevalence of obesity was 11.1, 24.8, 33.5, 38.0, and 36.0% for Asians (using standard cut points), Whites, Hispanics, Blacks, and other races, respectively; the prevalence of overweight was 32.6, 36.1, 39.0, 33.1, and 37.1% for Asians (using standard cut points), Whites, Hispanics, Blacks, and other races, respectively. Using WHO Asian cut points for obesity, 23.3% of Asians were identified to be obese and 40.0% overweight; these prevalence estimates are much higher than those using standard cut points. Regardless of using either cut points for obesity, male Asians had a higher prevalence than female Asians (13.4% versus 9.0%, standard cut points; 28.0% versus 19.2%, WHO Asian cut points; respectively, both *p* < 0.05). Among males, the prevalence of obesity was highest among other races (37.5%), followed by Hispanics (34.8%), Blacks (34.0%), and Asians (28.0%; WHO Asian cut points), and Whites (25.2%) (*p* < 0.0001). Among females, the prevalence of obesity was highest among Blacks (41.6%), followed by other races (33.4%), Hispanics (32.1%), Whites (24.5%), and Asians (19.2%; WHO Asian cut points) (*p* < 0.0001).

**Table 1:** Weighted prevalence of weight status in U.S. adults by sex and race/ethnicity, CHIS 2013–2014 (*n* = 47,970)

	Weighted prevalence by weight status, n (%)				
	Underweight	Normal weight	Overweight	Obesity	*P*-value
	(*n* = 748)	(*n* = 16,213)	(*n* = 17,501)	(*n* = 13,508)	
Asians (standard BMI cut points)					
Both sexes	136 (2.5)	2154 (53.8)	1166 (32.6)	354 (11.1)	< 0.0001
Males	29 (0.9)	834 (44.4)	637 (41.3)	173 (13.4)	< 0.0001
Females	107 (4.0)	1320 (62.1)	529 (24.9)	181 (9.0)	
Asians (WHO Asian BMI cut points)					
Both sexes	131 (2.4)	1371 (34.3)	1530 (40.0)	778 (23.3)	< 0.0001
Males	29 (0.9)	446 (22.3)	817 (48.9)	381 (28.0)	< 0.0001
Females	102 (3.7)	925 (44.9)	713 (32.2)	397 (19.2)	
Whites					
Both sexes	470 (1.4)	11,335 (37.6)	10,935 (36.1)	7865 (24.8)	< 0.0001
Males	99 (0.9)	3692 (32.3)	5246 (41.6)	3234 (25.2)	< 0.0001
Females	371 (1.9)	7643 (42.5)	5689 (31.3)	4631 (24.5)	
Hispanics					
Both sexes	84 (0.8)	2094 (26.7)	2991 (39.0)	2827 (33.5)	< 0.0001
Males	29 (0.7)	692 (22.3)	1375 (42.2)	1128 (34.8)	< 0.0001
Females	55 (1.0)	1402 (30.9)	1616 (35.9)	1699 (32.1)	
Blacks					
Both sexes	29 (1.4)	525 (27.5)	749 (33.1)	775 (38.0)	< 0.0001
Males	12 (1.2)	194 (26.4)	320 (38.3)	272 (34.0)	0.0899
Females	17 (1.6)	331 (28.4)	429 (28.4)	503 (41.6)	
Other races					
Both sexes	34 (0.5)	888 (26.5)	1296 (37.1)	1263 (36.0)	< 0.0001
Males	14 (0.4)	324 (22.0)	638 (40.1)	550 (37.5)	0.0002
Females	20 (0.6)	564 (31.8)	658 (33.4)	731 (33.4)	

WHO Asians BMI cut points were used for obesity among Asian Groups. Standard BMI cut off points were used for obesity among Whites, Hispanics, and Blacks

### Prevalence of obesity in Asian adults of California

The demographic and lifestyle characteristics among Californian Asians with obesity are described in Table 2. Overall, the obesity prevalence was higher in Asians who were males (*p* = 0.0012), and were 45–64 years old (*p* = 0.0054). Among Koreans, and Filipinos, males had higher obesity prevalence than females (20.7 vs. 6.7%, *p* = 0.0071; 43.7% vs. 24.5%, *p* = 0.0022, respectively). The highest prevalence of obesity was observed in age group 45–64 years old among Filipinos and Vietnamese as compared to that in age groups 18–44 years and 65 years or above (39.5% vs. 35.8 and 15.0%, *p* = 0.0063; 30.3% vs. 7.8 and 21.4%, *p* = 0.0017, respectively). For smoking status, Japanese had lower obesity prevalence in those who were current smokers than those who were not (7.4% vs. 27.5%, *p* = 0.0114). For marital status, compared to “other marital patterns” and “never married”, the prevalence of obesity for those who were married was highest among Vietnamese (12.9 and 8.8% vs. 23.8%, *p* = 0.0144). Obesity prevalence was lower in those with physical activity than those without physical activity in Vietnamese (13.7% vs. 32.5%, *p* = 0.0492). The prevalence of obesity was particularly high in Chinese who consumed more frequent fast food (31.2% for ≥3 times, 17.1% for 1–2 times, and 12.8% for never, *p* = 0.0046).

**Table 2:** Demographic and lifestyle characteristics among Californian Asians with obesity, CHIS 2013–2014 (*n* = 1441)

	All Asians n (%)	Chinese n (%)	Korean n (%)	Japanese n (%)	Filipino n (%)	Vietnamese n (%)
Overall sample size	*N* = 778	*N* = 180	*N* = 60	*N* = 153	*N* = 197	*N* = 73
**Sex**						
Male	381 (28.0)	84 (21.1)	28 (20.7)	75 (34.5)	98 (43.7)	32 (15.4)
Female	397 (19.2)	96 (14.3)	32 (6.7)	78 (21.5)	99 (24.5)	41 (20.2)
*P*-value	0.0012	0.1025	0.0071	0.0754	0.0022	0.5324
**Age**						
18–44 years	234 (22.8)	56 (17.8)	12 (15.0)	26 (21.6)	74 (35.8)	10 (7.8)
45–64 years	327 (27.4)	84 (17.0)	19 (9.8)	70 (31.0)	81 (39.5)	29 (30.3)
65 years or above	217 (16.1)	40 (15.1)	29 (11.4)	57 (28.3)	42 (15.0)	34 (21.4)
*P*-value	0.0054	0.9100	0.6487	0.7584	0.0063	0.0017
**Family income**						
< 100% FPL	126 (18.8)	28 (15.4)	16 (21.8)	12 (21.0)	20 (27.6)	30 (11.9)
100–299% FPL	229 (25.2)	58 (19.1)	26 (13.4)	28 (25.3)	70 (35.1)	27 (22.9)
≧300% FPL	423 (23.4)	94 (16.7)	18 (8.8)	113 (27.2)	107 (34.6)	16 (17.7)
*P*-value	0.2795	0.8095	0.2824	0.9058	0.7600	0.4792
**Smoking status**						
Current smoker	65 (27.1)	10 (28.0)	4 (12.7)	11 (7.4)	23 (43.4)	6 (14.7)
Not current smoker	713 (22.9)	170 (16.2)	56 (12.8)	142 (27.5)	174 (32.7)	67 (18.2)
*P*-value	0.3403	0.1422	0.9844	0.0114	0.2648	0.7638
**Marital status**						
Married	448 (22.6)	110 (16.9)	33 (10.5)	85 (28.8)	90 (31.0)	45 (23.8)
Others	147 (23.4)	28 (16.2)	17 (4.5)	27 (25.2)	48 (35.4)	17 (12.9)
Never married	183 (24.4)	42 (18.4)	10 (20.7)	41 (23.5)	59 (36.6)	11 (8.8)
*P*-value	0.8103	0.9270	0.0385	0.7787	0.7064	0.0144
**Education**						
High school	204 (21.1)	46 (13.6)	22 (15.7)	30 (31.0)	38 (30.7)	49 (14.9)
College	433 (26.1)	92 (19.8)	32 (13.3)	90 (24.9)	140 (32.6)	19 (24.3)
Graduate	141 (18.9)	42 (17.0)	6 (6.0)	33 (25.6)	19 (45.6)	5 (7.0)
*P*-value	0.0707	0.4335	0.4098	0.7629	0.3643	0.1717
**Physical activity**						
Yes	611 (22.7)	135 (16.9)	49 (12.5)	107 (24.2)	163 (33.7)	58 (13.7)
No	167 (26.0)	45 (18.7)	11 (15.0)	46 (35.5)	34 (34.3)	15 (32.5)
*P*-value	0.3041	0.7134	0.7367	0.1644	0.9436	0.0492
**Fast food consumption**						
Never	337 (21.8)	80 (12.8)	29 (8.0)	53 (25.9)	73 (43.7)	54 (21.4)
1–2 times	303 (22.2)	67 (17.1)	24 (15.1)	70 (21.5)	80 (28.3)	17 (19.0)
≧3 times	138 (29.1)	33 (31.2)	7 (19.7)	30 (37.4)	44 (28.4)	2 (2.4)
*P*-value	0.1096	0.0046	0.4043	0.3107	0.0942	0.0967

The Chi-square test was used to compare the prevalence of variables across race groups

*Abbreviations*: *CHIS* California Health Information Survey, *FPL* federal poverty level, *SD* standard deviation, *NA* not applicable

### Weighted logistic regression analyses in California adults

Table 3 shows results from multiple logistic regression analyses for the association between race/ethnicity and obesity among Californian adults. After adjusting for other factors, compared to Whites, being Hispanics and Blacks were associated with higher odds of obesity (OR = 1.47, 95% CI = 1.31–1.65; OR = 2.04, 95% CI = 1.65–2.53, respectively); being Chinese, Korean, and Vietnamese were associated with lower odds of obesity (OR = 0.28, 95% CI = 0.18–0.45; OR = 0.14, 95% CI = 0.04–0.46; OR = 0.28, 95% CI = 0.14–0.58, respectively). Furthermore, being aged 45–64 years of age (vs. 18–44 years of age), being male, having lower family income, married or other marital status, lower education level, lack of physical activity, and higher frequency of fast food consumption were associated with higher prevalence of obesity. When stratified by sex, among males, being Hispanic and Blacks were positively associated with obesity while being Chinese and Vietnamese were negatively associated with obesity; among females, being Hispanics and Blacks were positively associated with obesity, while being Chinese, Korean, Japanese, and Filipino were negatively associated with obesity.

**Table 3:** Multiple logistic regression analyses for the association between race/ethnicity and obesity among Californian adults, CHIS 2013–2014 (*n* = 47,970)

Variables	All OR (95% CI)	Male OR (95% CI)	Female OR (95% CI)
Race/ethnicity			
Whites (ref)			
Hispanics	1.47 (1.31–1.65)	1.63 (1.38–1.92)	1.36 (1.15–1.59)
Chinese	0.28 (0.18–0.45)	0.43 (0.25–0.75)	0.17 (0.07–0.39)
Korean	0.14 (0.04–0.46)	0.23 (0.03–1.62)	0.07 (0.03–0.20)
Japanese	0.58 (0.32–1.06)	0.70 (0.17–2.88)	0.54 (0.29–0.98)
Filipino	0.70 (0.45–1.08)	1.17 (0.65–2.08)	0.38 (0.20–0.72)
Vietnamese	0.28 (0.14–0.58)	0.07 (0.02–0.20)	0.55 (0.25–1.23)
Blacks	2.04 (1.65–2.53)	1.78 (1.33–2.40)	2.24 (1.67–3.00)
Other races	1.15 (0.88–1.51)	1.07 (0.75–1.51)	1.23 (0.83–1.80)
Age			
18–44 years (Ref)			
45–64 years	1.60 (1.41–1.82)	1.55 (1.28–1.88)	1.67 (1.39–2.01)
65 years or above	1.09 (0.95–1.25)	0.96 (0.78–1.19)	1.22 (1.00–1.48)
Sex			
Female (ref)			
Male	1.12 (1.02–1.23)	NA	NA
Family income			
≧300% FPL (ref)			
< 100% FPL	1.32 (1.12–1.57)	0.86 (0.66–1.13)	1.86 (1.49–2.31)
100–299% FPL	1.25 (1.11–1.40)	1.05 (0.91–1.23)	1.48 (1.25–1.76)
Smoking status			
Not current smoker (ref)			
Current smoker	0.89 (0.76–1.03)	0.77 (0.63–0.96)	1.08 (0.85–1.38)
Marital status			
Never married (ref)			
Married	1.32 (1.13–1.54)	1.46 (1.16–1.84)	1.20 (0.96–1.49)
Others	1.44 (1.25–1.65)	1.47 (1.18–1.85)	1.36 (1.09–1.69)
Education			
Graduate (ref)			
High school	1.31 (1.11–1.55)	1.36 (1.07–1.71)	1.27 (0.99–1.62)
College	1.18 (1.01–1.38)	1.17 (0.96–1.43)	1.15 (0.91–1.46)
Physical activity			
No (ref)			
Yes	0.65 (0.58–0.73)	0.68 (0.57–0.80)	0.64 (0.54–0.75)
Fast food consumption			
Never (ref)			
1–2 times	1.42 (1.27–1.58)	1.28 (1.07–1.52)	1.57 (1.36–1.80)
≧3 times	1.60 (1.38–1.86)	1.37 (1.12–1.68)	1.92 (1.56–2.37)

*Abbreviations*: *CHIS* California Health Information Survey, *CI* confidence interval, *NA* not applicable, *FPL* federal poverty level, *OR* odd ratio

### Weighted logistic regression analyses in Asian adults in California

Table 4 shows results from multiple logistic regression analyses for the association between race/ethnicity and obesity among Asian Americans. Overall, compared to Chinese, being Japanese and Filipino were associated with higher prevalence of obesity (OR = 2.75, 95% CI = 1.52–4.95; OR = 2.90, 95% CI = 1.87–4.49). When stratified by sex, similar findings were observed. In addition, being 65 years or above (vs. 18–44 years of age) for males was negatively associated with obesity (OR = 0.36, 95%% CI = 0.16–0.78), and being 45–64 years of age (vs. 18–44 years of age) for females was positively associated with obesity (OR = 2.14, 95% CI = 1.13–4.03).

**Table 4:** Multiple logistic regression analyses for the association between race/ethnicity and obesity among Asian Americans, CHIS 2013–2014 (*n* = 3810)

Variables	All, OR (95% CI)	Male, OR (95% CI)	Female, OR (95% CI)
Race/ethnicity			
Chinese (Ref)			
Korean	0.63 (0.31–1.29)	0.95 (0.38–2.39)	0.34 (0.11–1.07)
Japanese	2.75 (1.52–4.95)	2.89 (1.15–7.23)	2.46 (1.18–5.13)
Filipino	2.90 (1.87–4.49)	3.18 (1.68–6.01)	2.54 (1.25–5.17)
Vietnamese	1.11 (0.62–1.99)	0.76 (0.33–1.74)	1.78 (0.76–4.13)
Age			
18–44 years (Ref)			
45–64 years	1.34 (0.89–2.03)	0.94 (0.49–1.81)	2.14 (1.13–4.03)
65 years or above	0.70 (0.42–1.17)	0.36 (0.16–0.78)	1.38 (0.62–3.07)
Sex			
Female (ref)			
Male	1.91 (1.36–2.70)	NA	NA
Family income			
≧300% FPL (ref)			
< 100% FPL	0.94 (0.51–1.74)	0.76 (0.31–1.89)	1.12 (0.49–2.57)
100–299% FPL	1.16 (0.77–1.75)	1.12 (0.63–1.98)	1.35 (0.71–2.56)
Smoking status			
Not current smoker (ref)			
Current smoker	1.10 (0.60–2.01)	1.02 (0.50–2.07)	1.27 (0.29–5.51)
Marital status			
Never married (ref)			
Married	0.88 (0.53–1.45)	0.94 (0.48–1.84)	0.90 (0.40–2.03)
Others	0.67 (0.37–1.21)	0.71 (0.29–1.75)	0.49 (0.21–1.17)
Education			
Graduate (ref)			
High school	0.96 (0.52–1.76)	0.89 (0.42–1.89)	0.84 (0.33–2.13)
College	0.97 (0.57–1.62)	1.55 (0.75–3.19)	0.52 (0.24–1.15)
Physical activity			
No (ref)			
Yes	0.82 (0.54–1.25)	0.84 (0.46–1.53)	0.72 (0.41–1.26)
Fast food consumption			
Never (ref)			
1–2 times	0.73 (0.48–1.09)	0.55 (0.31–0.98)	1.19 (0.61–2.23)
≧3 times	0.76 (0.44–1.32)	0.57 (0.28–1.14)	1.16 (0.43–3.14)

*Abbreviations*: *CHIS* California Health Information Survey, *CI* confidence interval, *NA* not applicable, *FPL* federal poverty level, *OR* odd ratio

## Discussion

National surveys such as the National Health and Nutrition Examination Survey show that compared to other racial/ethnic groups Asian-Americans have lower obesity rates in the U.S. [21]. A recent study using CHIS has observed considerable ethnic disparity in adult obesity prevalence in California: African Americans (36.1%), Latinos (33.6%), Whites (22.0%), and Asians (9.8%). The prevalence in African Americans was three times higher than that in Asians [12]. However, it has been criticized that such national or state surveys have not considered the differences between ethnic groups in Asians [21]. This study assessed differences between the prevalence of obesity in Asian racial/ethnic groups, using definitions by standard cut points and WHO Asian cut points. It was found that overall the prevalence of obesity was 11.1 and 32.6% for overweight (using standard cut points). In contrast, using the WHO Asian cut points for obesity, the prevalence of obesity was 23.3% and the prevalence of overweight was 40.0% among Asian Americans; these estimates are much higher compared to those using standard cut points. The reason as to why Asians have lower BMI rates than Whites with adjustment for covariates remains unclear [9]. For the same age, sex, and percentage of body fat, BMI is consistently lower in Asians than in Whites by about 2–3 kg/m^2^. This is because body build and muscularity may be different in these populations [22]. Furthermore, absolute or relative metabolic risk cannot be corresponded similarly in all ethnic groups using standard cut points for obesity [19]. Because of these limitations of BMI measure in Asian populations, lower BMI cut points for Asians have been proposed by WHO using all available data from Asian countries; for Asians, overweight was defined as a BMI of 23–27.5 kg/m^2^ and obesity as a BMI of ≥27.5 kg/m^2^ [19]. Due to the possibility that lifestyles could be very different between Asians living in their original countries and Asians who have immigrated to live in Western countries (e.g., the U.S.), whether these international guidelines for Asians are appropriate for Asian Americans has been subject to discussion [16].

Although there was a debate on whether Asian specific BMI cut points could be used for all Asians worldwide particularly in Western countries [16, 23–26], a number of studies have been conducted for addressing this issue; these studies found that, compared to other racial/ethnic groups, Asian Americans have lower rates of overweight/obesity [21, 27, 28], but they have a higher risk of type 2 diabetes, hypertension, and associated metabolic abnormalities [14, 29–32]. Thus, it appears not appropriate to use standard BMI cut points to examine the prevalence of obesity among Asians Americans, because its impact in these populations may be underestimated. Instead, applying the WHO Asian BMI cut points may have some benefits, e.g., 1) having better estimates of health conditions attributable to obesity [16, 29, 30] and 2) having significant clinical implication in identifying at-risk Asian Americans [33].

This study found that in California, the prevalence of obesity was 23.3, 24.8, 33.5, 38.0, and 36.0% for Asians, Whites, Hispanics, Blacks, and other races, respectively. After adjusting for other factors, compared to Whites, being Hispanics and Blacks were associated with a higher prevalence of obesity; being Chinese, Korean, and Vietnamese were associated with lower prevalence of obesity. This finding is consistent with a previous study that examined racial/ethnic disparities in obesity among adults in California using the 2003 CHIS, where the prevalence of obesity was highest in Blacks, followed by Hispanics, Whites, and Asians. Reasons that might explain racial/ethnic differences in obesity may be related to a number of factors that are correlated to both race/ethnicity and BMI, e.g., different SES, demographic characteristics, and behavioral factors [9, 10]. For example, Hispanics in California have lower educational attainment and higher poverty concentrations than other ethnic groups; Blacks have higher poverty rates than whites and more than a third have not attended college, and they are more likely than other groups to report no walking [9].

Among Asians in California, the obesity prevalence was higher in those who were males, and were 45–64 years old. It is interesting to observe a higher prevalence of obesity in males among Asians, which is different from that among other races. For example, previous studies found that overall there are more obese women than men. In developing countries (e.g., in the Middle East and North Africa), such disparities are more obvious among women [34]. In the U.S., there was no difference in overall obesity prevalence by sex in 2011–2012. However, sex difference in obesity is observed among non-Hispanic black adults (56.6% for women vs. 37.1% for men) [28]. Among Asian Americans, women likely pay more attention to healthier lifestyle, e.g., engaging in more physical activity, and thus are less likely to be obese.

Asian Americans were the fastest growing race/ethnic group in the U.S. in the past decade, and these numbers will continue to rise in the coming decades. Asian Americans are expected to double in population size with a projected increase to more than 43 million by 2050 in the U.S. [12]. California has the largest Asian population in the U.S. [35]. Compared to 2001, obesity prevalence in 2011–2012 has increased among Chinese (3.8% versus 6.1%), Japanese (9.0% versus 15.0%), Filipino (8.8% versus 18.7%), and Vietnamese (3.4% versus 6.8%). However, it has not changed significantly among Korean (1.5% versus 2.1%) [2]. The present study found that compared to Chinese, being Japanese or Filipino was associated with a higher prevalence of obesity, which is consistent with previous findings [2]. In addition, another report also showed that among Asian adults, Filipino adults (14%) were more than twice as likely to be obese as Asian Indian (6%), Vietnamese (5%), or Chinese adults (4%), and were 70% more likely to be obese as compared to the overall Asian population [36]. It is important to investigate the obesity disparity in Asians, because they have pronounced socioeconomic disparities across ethnic groups, with some ethnic groups’ SES far exceeding national averages, while some other groups’ SES being lowest in the U.S. [15].

This study had some limitations. First, the data were collected almost 8 years ago; as such, the data may not present the current demographic composition in California as the latter has changed in the last decade. Second, data were collected by self-report, making responses prone to social desirability bias and recall bias. Third, although not necessarily a limitation, our findings may not be generalizable to Asian populations in other states. Fourth, because this study was cross-sectional, the directionality of cause and effect of the association between race/ethnicity and obesity cannot be established. Thus, further studies need to use longitudinal data to explore their causal relationships. However, CHIS has large sample sizes, even for subgroups, and results are applicable broadly to the adult population in the United States, especially for Asian population. To date, few obesity reports in California have provided important information regarding obesity among adults in the state; the present study offers new insights into obesity research among Asian Americans residing in California.

## Conclusion

The prevalence of obesity was found to be higher in adult Asian Americans in California by using the Asian-specific standards than previously acknowledged. Further, ethnic/racial disparities in Asian Americans in California were observed in 2013–2014. Compared to Whites, being Chinese, Korean, and Vietnamese were associated with a lower prevalence of obesity. Among Asians, compared to Chinese, being Japanese and being Filipino were associated with higher prevalence of obesity. These findings can help design more effective interventions to reduce racial and ethnic disparities in obesity, especially for Asian Americans.

## Data Availability

The California Health Interview Survey data that support the findings of this study are available from https://healthpolicy.ucla.edu/chis/data/Pages/public-use-data.aspx. The Public Use Data files in CHIS is publicly available to anyone who registers in CHIS website.

## Abbreviations

BMI: Body mass index
CHIS: California Health Interview Survey
CI: Confidence interval
FPL: Federal poverty level
OR: Odds ratio
RDD: Random-digit-dial

## References

[1] Flegal K, Moran-Kruszon D, Carroll M (2016). Trends in obesity among adults in the United States, 2005-2014. JAMA..

[2] Wolstein J, Babey SH, Diamant AL. Obesity in California. Los Angeles: UCLA Center for Health Policy Research; 2015. Retrieved from http://www.healthpolicy.ucla.edu/publications/Documents/PDF/2015/obesityreport-jun2015.pdf.

[3] Griffith DM, Johnson-Lawrence V, Gunter K, Neighbors HW (2011). Race, SES, and obesity among men. Race Soc Probl.

[4] Malnick SD, Knobler H (2006). The medical complications of obesity. QJM..

[5] Kelley EA, Bowie JV, Griffith DM, Bruce M, Hill S, Thorpe RJ (2016). Geography, race/ethnicity, and obesity among men in the United States. Am J Mens Health.

[6] Ogden CL, Carroll MD, Kit BK, Flegal KM (2014). Prevalence of childhood and adult obesity in the United States, 2011-2012. JAMA..

[7] Hales CM, Carroll MD, Fryar CD, Ogden CL (2020). Prevalence of obesity and severe obesity among adults: United States, 2017–2018. NCHS Data Brief, no 360.

[8] Singh GK, Lin SC. Dramatic increases in obesity and overweight prevalence among Asian subgroups in the United States, 1992-2011. ISRN Prev Med. 2013;2013:898691. 10.5402/2013/898691. 10.5402/2013/898691 PMC4045452 24967142

[9] Lee H. Obesity among California adults: racial and ethnic differences. p.;cm. "August 8, 2016". Public Policy Institute of California; 2006. ISBN-13: 978-1-58213-122-1.

[10] Reyes B (2001). A portrait of race and ethnicity in california: an assessment of social and economic well-being.

[11] Chang V, Christakis N (2005). Income inequality and weight status in U.S. metropolitan areas. Soc Sci Med.

[12] Yi SS, Kwon SC, Wyatt L, Islam N, Trinh-Shevrin C (2015). Weighing in on the hidden Asian American obesity epidemic. Prev Med.

[13] Cook WK, Tseng W, Bautista R, John I (2016). Ethnicity, socioeconomic status, and overweight in Asian American adolescents. Prev Med Rep.

[14] Zhou M, Xiong YS (2005). The multifaceted American experiences of the children of Asian immigrants: lessons for segmented assimilation. Ethn Racial Stud.

[15] Cook WK, Chung C, Tseng W (2011). Demographic and socioeconomic profiles of Asian Americans, native Hawaiians, and Pacific Islanders.

[16] Palaniappan LP, Wong EC, Shin JJ, Fortmann SP, Lauderdale DS (2011). Asian Americans have greater prevalence of metabolic syndrome despite lower body mass index. Int J Obes.

[17] Staimez LR, Weber MB, Narayan KM, Oza-Frank R (2013). A systematic review of overweight, obesity, and type 2 diabetes among Asian American subgroups. Curr Diabetes Rev.

[18] California Health Interview Survey (CHIS) (2016). CHIS 2013–2014 Methodology Report Series.

[19] WHO Expert Consultation (2004). Appropriate body-mass index for Asian populations and its implications for policy and intervention strategies. Lancet..

[20] UCLA Center for Health Policy Research. AskCHIS 2013-2014. California Health Interview Survey (CHIS) 2013-2014. Adult questionnaire. Version 5.4. January 8, 2015. Access in 2016 at http://askchisne.ucla.edu/chis/design/Documents/chis2013adultquestionnaire.pdf. Accessed 10 Mar 2016.

[21] Wang Y, Beydoun MA (2007). The obesity epidemic in the United States- gender, age, socioeconomic, racial/ethnic, and geographic characteristics: a systematic review and meta-regression analysis. Epidemiol Rev.

[22] Deurenberg-Yap M, Chew SK, Deurenberg P (2002). Elevated body fat percentage and cardiovascular risks at low body mass index levels among Singaporean Chinese, Malays and Indians. Obes Rev.

[23] Low S, Chin MC, Ma S, Heng D, Deurenberg-Yap M (2009). Rationale for redefining obesity in Asians. Ann Acad Med Singap.

[24] Pan WH, Yeh WT (2008). How to define obesity? Evidence-based multiple action points for public awareness, screening, and treatment: an extension of Asian-Pacific recommendations. Asia Pac J Clin Nutr.

[25] Razak F, Anand SS, Shannon H, Vuksan V, Davis B, Jacobs R, Teo KK, McQueen M, Yusuf S (2007). Defining obesity cut points in a multiethnic population. Circulation..

[26] Stevens J (2003). Ethnic-specific revisions of body mass index cutoffs to define overweight and obesity in Asians are not warranted. Int J Obes Relat Metab Disord.

[27] Bates LM, Acevedo-Garcia D, Alegria M, Krieger N (2008). Immigration and generational trends in body mass index and obesity in the United States: results of the National Latino and Asian American survey, 2002–2003. Am J Public Health.

[28] Ogden CL, Carroll MD, Kit BK, Flegal KM (2013). Prevalence of obesity among adults: United States, 2011–2012.

[29] Karter AJ, Schillinger D, Adams AS, Moffet HH, Liu J, Adler NE, Kanaya AM (2013). Elevated rates of diabetes in Pacific islanders and Asian subgroups: the diabetes study of northern California (DISTANCE). Diabetes Care.

[30] King GL, McNeely MJ, Thorpe LE (2012). Understanding and addressing unique needs of diabetes in Asian Americans, native Hawaiians, and Pacific islanders. Diabetes Care.

[31] Lee JW, Brancati FL, Yeh HC (2011). Trends in the prevalence of type 2 diabetes in Asians versus whites: results from the United States National Health Interview Survey, 1997–2008. Diabetes Care.

[32] Wong RJ, Chou C, Sinha SR, Kamal A, Ahmed A (2014). Ethnic disparities in the association of body mass index with the risk of hypertension and diabetes. J Community Health.

[33] Jih J, Mukherjea A, Vittinghoff E, Nguyen TT, Tsoh JY, Fukuoka Y, Bender MS, Tseng W, Kanaya AM (2014). Using appropriate body mass index cut points for overweigt and obesity among Asian Americans. Prev Med.

[34] Kanter R, Caballero B (2012). Global gender disparities in obesity: a review. Adv Nutr.

[35] U.S. Census Bureau. FFF: Asian/Pacific American Heritage Month: May 2016. Facts for Features. Retrieved from http://www.census.gov/newsroom/facts-for-features/2016/cb16-ff07.html. Accessed 20 Apr 2020.

[36] Barnes, et al. Health characteristics of the Asian adult population: United States, 2004–2008. http://www.cdc.gov/nchs/data/ad/ad394.pdf. 18271366

